# Quercetin ingestion modifies human motor unit firing patterns and muscle contractile properties

**DOI:** 10.1007/s00221-021-06085-w

**Published:** 2021-03-19

**Authors:** Kohei Watanabe, Aleš Holobar

**Affiliations:** 1 Laboratory of Neuromuscular Biomechanics, Faculty of Liberal Studies and Sciences and School of International Liberal Studies, Chukyo University, Showa-ku, YagotohonmachiNagoya, 466-8666 Japan; 2 Faculty of Electrical Engineering and Computer Science, University of Maribor, Maribor, Slovenia

**Keywords:** Ergogenic aids, Nutritional supplementation, Multichannel surface electromyography, Motor unit identification, Quercetin

## Abstract

Quercetin is a polyphenolic flavonoid that has reported to block the binding of adenosine to A1 receptors at central nervous system and increase calcium release from the sarcoplasmic reticulum at skeletal muscle. The aim of the present study was to investigate the acute effect of quercetin ingestion on motor unit activation and muscle contractile properties. High-density surface electromyography during submaximal contractions and electrically elicited contraction torque in knee extensor muscles were measured before (PRE) and 60 min after (POST) quercetin glycosides or placebo ingestions in 13 young males. Individual motor units of the vastus lateralis muscle were identified from high-density surface electromyography by the Convolution Kernel Compensation technique. Firing rates of motor units recruited at 30–50% of the maximal voluntary contraction torque (MVC) were increased from PRE to POST only with quercetin (9.0 ± 2.3 to 10.5 ± 2.0 pps, *p* = 0.034). Twitch torque during doublet stimulation was decreased from PRE to POST with placebo (77.1 ± 17.1 to 73.9 ± 17.6 Nm, *p* = 0.005), but not with quercetin (*p* > 0.05). For motor units recruited at < 10% of MVC, normalized firing rate were decreased with quercetin (1.52 ± 0.33 to 1.58 ± 0.35%MVC/pps, *p* = 0.002) but increased with placebo (1.61 ± 0.32 to 1.57 ± 0.31%MVC/pps, *p* = 0.005). These results suggest that ingested quercetin has the functional roles to: mitigate reduction in the muscle contractile properties, enhance activations of relatively higher recruitment threshold motor units, and inhibit activation of relatively lower recruitment threshold motor units.

## Introduction

Quercetin is a polyphenolic flavonoid extractable from several plant foods such as tea, onions, apples, and dark green vegetables (Konrad and Nieman 2015), and it has been granted a GRAS status (generally recognized as safe) by the U. S. Food and Drug Administration (FDA). Based on extensive pre-clinical data, its anti-inflammatory and antioxidant activities, and ability to promote mitochondrial biogenesis have attracted interest regarding its usage as an ergogenic aid to enhance physical performance and/or improve health (Davis et al. 2009). On the other hand, quercetin has other interesting effects on the neuromuscular system (Lee et al. 2002; Cheuvront et al. 2009; Davis et al. 2009).

Various flavonoids exhibit adenosine A_1_ receptor antagonist activity and quercetin showed the highest affinity among various flavonoids for this receptor (Alexander 2006). Therefore, quercetin is recognized as having the role of blocking the binding of adenosine to A_1_ receptors and promoting the release of neurotransmitters such as acetylcholine and dopamine (Cheuvront et al. 2009). Also, quercetin has actions increasing calcium release from the sarcoplasmic reticulum (Lee et al. 2002). Since these actions of quercetin on central and peripheral levels of the neuromuscular system (Lee et al. 2002; Alexander 2006) are similar to those of caffeine, which is the most commonly consumed ergogenic aid in the world (Graham 2001; McLellan et al. 2016; Grgic et al. 2019), quercetin has received attention as an ergogenic aid to alter arousal, motor unit activation, and muscle contractions and improve physical performance, similarly to caffeine.

Very few studies have been reported on the acute effect of quercetin on the human neuromuscular system (Patrizio et al. 2018; Bazzucchi et al. 2019). Patrizio et al. (2018) showed lower maximal voluntary contraction (MVC) reduction after a resistance training program with greater neuromuscular efficiency following 8 days of receiving a single dose of quercetin (Patrizio et al. 2018). This suggest that the participants could perform the training program with lower physiological burden when the quercetin was ingested. Because neuromuscular efficiency (Rainoldi et al. 2008) was defined as the ratio between the performed force and myoelectrical activation during MVC in this previous study, it is considered that quercetin ingestion improves excitation–contraction coupling in skeletal muscle. Bazzucchi et al. (2019) reported that 14 days of quercetin supplementation induced an increase of MVC and lower decay of muscle fiber conduction velocity following fatiguing eccentric contractions (Bazzucchi et al. 2019). Considering the possible mechanisms behind the ergogenic effects of quercetin (Lee et al. 2002; Alexander 2006) with similar pathways to those of caffeine (Lee et al. 2002; McLellan et al. 2016), alterations in neuromuscular efficiency (Patrizio et al. 2018) and muscle fiber conduction velocity (Bazzucchi et al. 2019) following quercetin ingestions could be explained by the effects of quercetin ingestions on central and peripheral levels, respectively. For example, quercetin ingestions may induce changes in motor unit firing/recruitment patterns and in muscle contractile properties due to its role as an adenosine receptor antagonist (Alexander 2006) and an increase in calcium release from the sarcoplasmic reticulum (Lee et al. 2002). However, these recent studies (Patrizio et al. 2018; Bazzucchi et al. 2019) did not localize the physiological adaptations to either the central or peripheral sites of the neuromusclar system, such as motor unit activation and muscle contractile properties.

The aim of the present study was to investigate the acute effect of quercetin ingestion on motor unit activation and muscle contractile properties. We employed motor unit identification based on high-density surface electromyography (HDsEMG) and electrical evoked twitch contraction to quantify motor unit activation and muscle contractile properties. We hypothesized that quercetin ingestion induces (1) alterations in the recruitment threshold-dependent motor unit firing rate, (2) greater evoked twitch torques, and (3) decrease in firing rate due to greater neuromuscular efficiency during voluntary contractions based on ergogenic effects of quercetin (Lee et al. 2002; Alexander 2006) and results of previous studies which were tested the effect of quercetin as short-term intervention (Patrizio et al. 2018; Bazzucchi et al. 2019).

## Materials and methods

### Participants

Thirteen healthy young males (mean ± SD: age: 23.6 ± 5.1 years, height: 170.2 ± 6.2 cm, body weight: 62.9 ± 11.5 kg) participated in this study. Participants who had any exercise restrictions imposed by medical doctors were excluded from the study. The subjects gave written informed consent for the study after receiving a detailed explanation of the purposes, potential benefits, and risks associated with participation. All procedures used in this study were approved by the Research Ethics Committee of Chukyo University (2019-003).

### Experimental design

Participants came to the laboratory on 2 days separated by at least 48 h and were randomly assigned the days for the trials with ingestions of quercetin or placebo. They were asked to refrain from vigorous-intensity exercise, consumptions of food or drinks containing quercetin and/or caffeine, and the ingestion of other possible ergogenic aids which are sold as functional foods 24 h before testing.

Following the preparations for experiments, the participants sat in a dynamometer and remained resting in that position for 5 min. Participants’ hip and knee joint angles were fixed at 170° and 90°, respectively, and the distal part of the shank of the right leg was fixed to the force transducer (LU-100KSE; Kyowa Electronic Instruments, Tokyo, Japan) in the dynamometer to measure the knee extension joint torque.

Under the resting condition, the tendon reflex (T-reflex) was applied to assess the responsiveness of the reflex pathway. Rapid maximal contraction and MVC of isometric knee extension were measured as the indicator of physiological responses including both central nervous system and peripheral muscle. Participants also performed submaximal ramp contractions for recording HDsEMG in order to identify individual motor units’ activation from the knee extensor muscles. Electrically elicited contraction of knee extensor muscles was conducted to quantify the contractile properties of muscles.

**Fig. 1:**
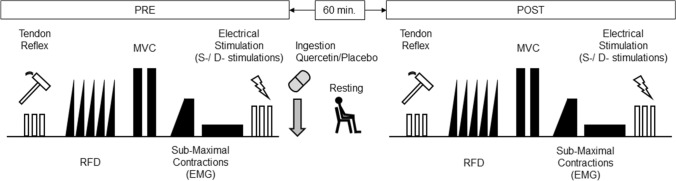
Schematic overview of the experimental protocol. RTD, rate of torque development; MVC, maximal voluntary contraction; EMG, electromyography; S-/D-stimulations, singlet and doublet stimulations. PRE, before ingestions; POST, 60 min after ingestions

After these measurements (PRE), the participants took six gelatin-coated and colored capsules containing 500 mg of quercetin glycosides with 2.0–2.5 g of dextrin (quercetin) or 0 mg of quercetin with 2.0–2.5 g of dextrin (placebo) with ~ 300 mL of water. Quercetin glycosides are more water soluble and bioavailable than quercetin aglycone, which does not exist in a glycoside or conjugate form (Makino et al. 2009). When absorbed, quercetin glycorandomsides are enzymatically converted into the aglycone form and have been found to exhibit beneficial effects similar to those of the corresponding quercetin aglycone. Quercetin glycosides were enzymatically manufactured at San-Ei Gen F.F.I., Inc. (Osaka, Japan) from isoquercitrin prepared from quercetin-3-O-rutinoside. In a GRAS statement from the FDA, up to 500 mg/serving of quercetin is acceptable. The capsules for quercetin glycosides and placebo had the same shape, color, and weight and randomized, double-blind, placebo-controlled treatment was administered in this study. Participants sat in a chair for 60 min after taking quercetin or the placebo and were asked to keep resting, and then, we performed the measurements (POST). The protocols are shown in Fig. 1. We used the same electrodes for surface EMG and electrical stimulation between PRE and POST on the same day, meaning that we did not replace them during the resting periods.

### Tendon-reflex

During the 5-min rest before the T-reflex test and while performing the test, participants wore blinding goggles and listened to relaxing music through headphones to restrict visual and auditory information. During the measurements, the knee of the right leg was tightly fixed to a dynamometer with a sponge and strap. A hand-made rubber-tipped hammer with electrical switch was used to tap the patellar tendon of the right leg. An equal amount of force was applied on the patellar tendon by the motion of a pendulum from the same height (1.025 J: kg∙m/s). Details of materials for measurements of T-reflex have been described in our previous study (Watanabe 2018).

**Fig. 2:**
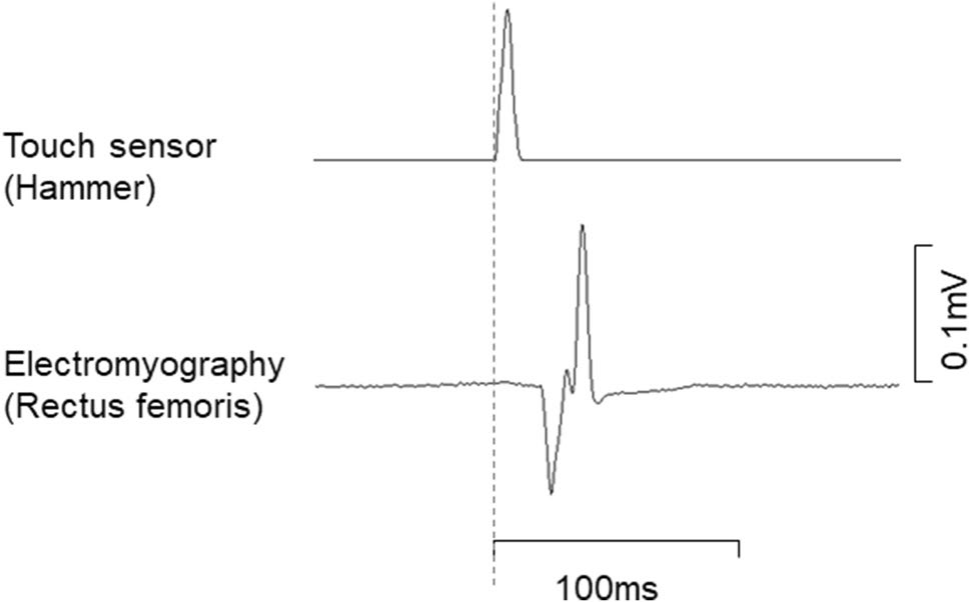
Representative data of electromyography during tendon reflex

We applied ten taps with random intervals of 5–10 s. During this measurement, a single bipolar surface EMG was recorded from the rectus femoris (RF) muscle. Electrodes for the RF muscle were located at the midpoint of the line between the anterior superior iliac spine and superior edge of the patella. Disposable Ag/AgCL circle-shaped electrodes (detection area of 1.5 cm in diameter) with a 2 cm inter-electrode distance (272S, Noraxon USA Inc., Arizona, USA) were attached on the RF muscle and connected to an amplifier (FA-DL-141, 4 assist, Tokyo, Japan). The reference electrode for surface EMG from the RF muscle was attached at the iliac crest. Surface EMG was sampled at 2000 Hz and recorded by an A-D converter (Power Lab 16/35, AD Instruments, Melbourne, Australia) with the electrical signal from the hammer and knee joint torque. Recorded surface EMG signals were filtered with an 8th order Bessel bandpass filter at 10–450 Hz and full-wave rectified. The peak absolute values of amplitude of the filtered and rectified surface EMG was detected within 100 ms from the timing of the tap as the patellar tendon reflex and averaged values from ten taps were used for further analysis (Fig. 2). This measurement process for the patellar tendon reflex was determined according to the methods and equipment used in our previous study (Watanabe 2018).

### RTD and MVC

The rate of torque development (RTD) and maximal voluntary contraction (MVC) during isometric knee extension of the right leg were measured, respectively. For RTD, the participants performed two sets of five contractions. Rest intervals between contractions and sets were 10 s and 2 min, respectively. Participants were instructed to perform the greatest torque as quickly as possible following a verbal count by the investigator during RTD trials. Maximal slope of torque–time curve was detected as RTD (Vila-Cha et al. 2010) and highest values from three of ten contractions were averaged for further analysis (Maffiuletti et al. 2016).

For MVC, the participants performed at least two MVCs with a 2-min rest interval between them. An MVC trial included a gradual increase in the knee extension force to maximum effort in 2–3 s, and the plateau phase at maximum effort was maintained for 2–3 s with a verbal count given at 1-s intervals. Prior to RTD and MVC measurements, participants performed 1–2 RTD and MVC trial at submaximal levels as familiarization and warm-up. RTD and MVC were calculated as products of the knee extension force measured by the force transducer and distance between the estimated rotation axes, i.e., the midpoint between the lateral femoral epicondyle and lateral head of fibula, for the knee joint in the sagittal plane. Averaged values of ten RTD trials and the highest MVC torque of two MVC trials were used for further analysis.

### Electrical stimulation

Contraction torques of knee extensor muscles during singlet and doublet stimulation were measured to estimate muscle contraction properties. Two electrodes (2 × 15 cm) were attached at proximal and distal sites of the right quadriceps femoris muscle (Watanabe et al. 2017). Electrodes were located at the center to lateral position to cover proximal regions of RF and VL muscles for the proximal site and at the center to medial position to cover distal regions of RF and vastus medialis muscles for distal sites. Electrical stimulation was applied via these electrodes using a constant current stimulator (DS7AH, Digitimer, Ltd., Hertfordshire, UK) with a 200 µs pulse width. At PRE, the current intensity for singlet stimulation was increased by 100 mA from 400 mA until maximal knee extension joint torque was achieved. This maximal current intensity at PRE was used for measurements of contraction torques during singlet and doublet stimulation at PRE and POST. The pulse gap for doublet stimulation was 100 ms. Elicited peak torques during one singlet and three doublet stimulation were measured. For doublet stimulation, the averaged value across three contractions was used for further analysis. Duration between electrical stimulation and occurrence of twitch torque and RTD was also calculated for singlet stimulations.

### Submaximal contractions and recording of high-density surface EMG

For recording HDsEMG signals, participants performed ramp contractions from 0 to 50% of MVC and sustained contractions at 10% of MVC during isometric knee extension. Ramp contraction consisted of a 17 s increasing phase from the baseline to 50% of the MVC force level with an approximately 3% MVC/sec rate of force increase and 10 s sustained phase at 50% of the MVC force level. Sustained contraction consisted of a 1–2 s increasing phase from the baseline to 10% of the MVC force level and a 120 s sustained phase at 10% of the MVC force level. The torque measurement system and posture during these submaximal contractions were the same as those during RTD and MVC. During these submaximal contractions, the performed and target torques were shown on the monitor of a personal computer as visual feedback. We used MVC at PRE to calculate the target forces for submaximal contractions, which means that the participants exerted the same absolute torque during submaximal contractions at PRE and POST on the same day.

HDsEMG signals were recorded from the vastus lateralis (VL) muscle with a semi-disposable adhesive grid of 64 electrodes and a 1-mm diameter and 8-mm inter-electrode distance (ELSCH064NM2, OT Bioelectronica, Torino, Italy). The electrodes were organized in 13 rows and 5 columns with 1 missing electrode in the upper left corner. The midpoint of the line between the head of the greater trochanter and inferior lateral edge of the patella was used as the center of the electrode grid, and the line was also used to determine the direction of electrode grids, whereby columns of electrodes were aligned along it. A reference electrode (WS1, OT Bioelectronica, Torino, Italy) was placed at the right knee. Monopolar surface EMG signals were recorded with a bandpass filter (10–500 Hz), and amplified by a factor of 150, sampled at 2048 Hz, and converted to a digital form by a 16-bit analog-to-digital converter (Quattrocento, OT Bioelectronica, Torino, Italy). The signal from the force transducer was synchronized with this analog-to-digital converter.

Recorded monopolar surface EMG signals were transferred to analysis software (MATLAB R2019a, MathWorks GK, Tokyo, Japan), and individual motor units were identified by the Convolution Kernel Compensation (CKC) technique using DEMUSE software (Holobar and Zazula 2004, 2008; Merletti et al. 2008; Holobar et al. 2009). We followed the decomposition procedure previously and extensively validated based on signals from various skeletal muscles (Holobar et al. 2009; Farina et al. 2010; Gallego et al. 2015a, b; Yavuz et al. 2015; Watanabe et al. 2016, 2018). The pulse-to-noise ratio (PNR), introduced by Holobar et al. (2014), was used as an indicator of the motor unit identification accuracy (Holobar et al. 2014), and only motor units with PNR > 30 dB (corresponding to an accuracy of motor unit firing identification > 90%) were used for further analysis; all other motor units were discarded (Holobar et al. 2014). After decomposition, discharge timings of individual motor units were independently examined by two experienced investigators. Discharge times for individual motor units were used to calculate instantaneous motor unit firing rates. We excluded discharges with inter-discharge intervals < 33.3 or > 250 ms, since firing rates calculated from this range of inter-discharge intervals are unusually high (> 30 Hz) or low (< 4 Hz) for the VL muscle. These procedures were the same as those used in our previous studies (Watanabe et al. 2016, 2020).

**Fig. 3:**
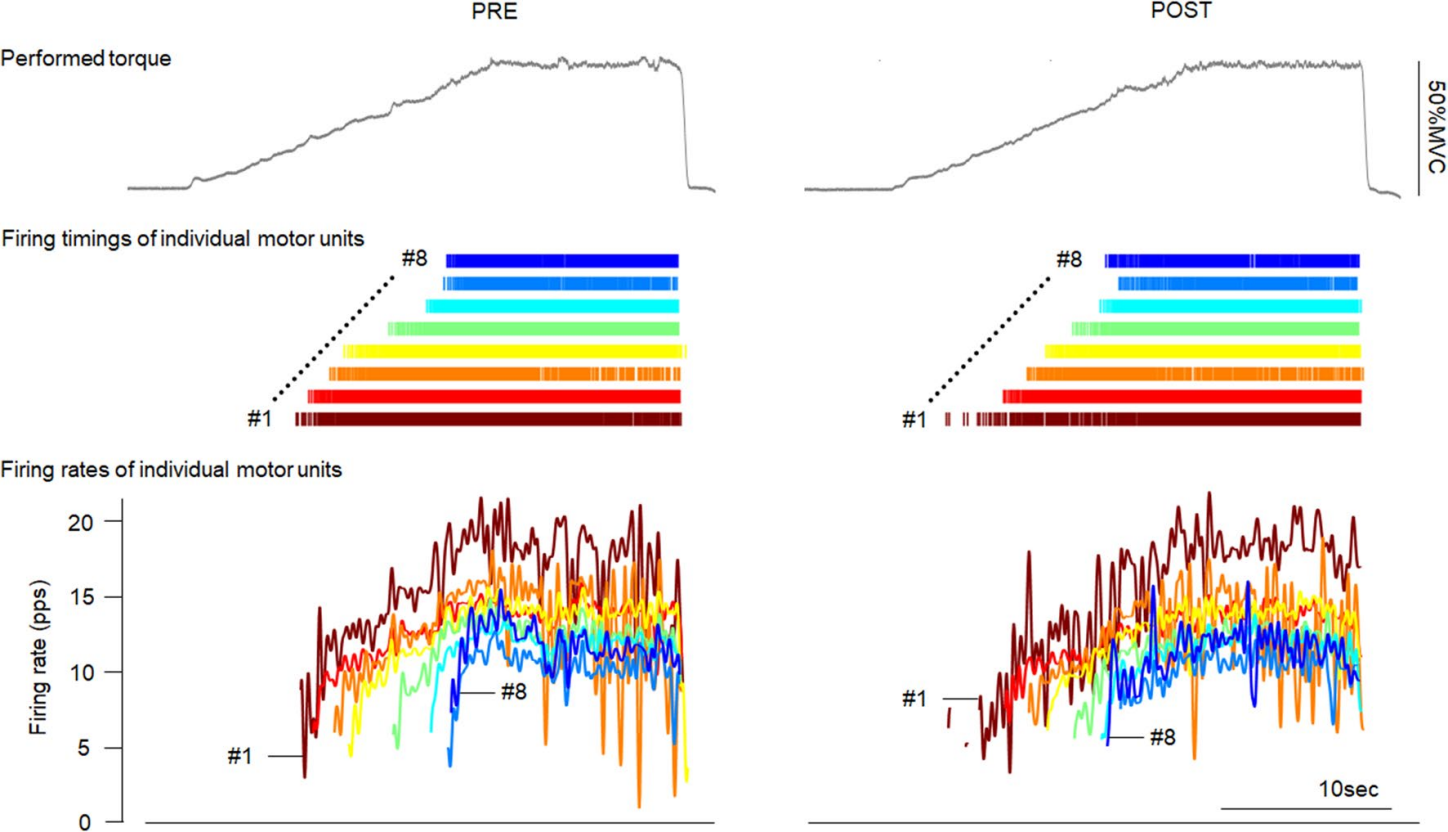
The performed torques (upper), firing timings (middle), and firing rates (bottom) of individual motor units before and after quercetin ingestion in a representative participant

Detected motor units were tracked between PRE and POST on the same day using the CKC technique to calculate the motor unit identification filters from HDsEMG signals at PRE and apply them to HDsEMG signals at POST (Fig. 3). Also, tracked motor units were assessed their similarity of the action potential waveform shape by cross-correlation analysis. This method allows very efficient tracking of individual motor unit firing patterns across different conditions, as previously demonstrated by Del Vecchio et al. (2019). The previously introduced criterion of PNR > 30 dB was also applied to motor unit tracking, ensuring an accuracy of motor unit firing identification > 90% at POST.

For ramp contractions, median values of firing rates of individual motor units were calculated from instantaneous firing rates for the ranges of 10–20% of MVC (15% of MVC), 20–30% of MVC (25% of MVC), 30–40% of MVC (35% of MVC), and 40–50% of MVC (45% of MVC) for ramp contractions. Detected motor units were divided into three motor unit groups by the recruitment threshold at PRE, i.e., motor units recruited at < 15% of MVC (MU10), 15–35% of MVC (MU25), and 35–45% of MVC (MU40), and firing rate analyses were performed for these motor unit groups. Recruitment thresholds of motor units were determined as the force level which the motor unit have first discharge with the above-mentioned criteria. For sustained contractions, median values of firing rates of individual motor units were calculated from instantaneous firing rates for the ranges 0–10 s (start), 50–60 s (middle), and 110–120 s (end) for sustained contractions. Motor unit firing rates with > 30% coefficient of variation were excluded from further analysis (Fuglevand et al. 1993). For sustained contractions, the rates of performed force and firing rate were calculated as normalized firing rate (pps/%MVC). Motor units detected during a trial for quercetin or placebo were merged across all participants and their mean values were used for further analysis.

### Statistics

The results are reported as the mean ± SD. Since our results included non-normal distributed data and were based on small samples, we used non-parametric statistical tests. To test the effect of quercetin or placebo ingestions, T-reflex, RTD, MVC, twitch torques during singlet and doublet stimulations, motor unit firing rates, normalized firing rate, and recruitment thresholds of motor units during ramp contractions were compared between PRE and POST for each day by the Wilcoxon signed rank test. T-reflex, RTD, MVC, twitch torques during singlet and doublet stimulations at PRE and POST were compared between quercetin and placebo ingestions by the Wilcoxon signed rank test. The proportions of PRE and POST for T-reflex, RTD, MVC, twitch torques during singlet and doublet stimulations were also compared by Wilcoxon signed rank test. Recruitment thresholds of the analyzed motor units during ramp contractions was compared between PRE and POST by the Wilcoxon signed rank test. To quantify the effect size, we calculated correlation coefficient from *Z*-score (r) and this value from 0 to 1 indicates no relationship to a perfect relationship (Hulley et al. 2013; Tomczak and Tomczak 2014). The level of significance on two groups comparison was set at 0.05. Statistical analysis was performed using SPSS (version 21.0, SPSS, Tokyo, Japan).

## Results

No significant changes were observed from PRE to POST in T-reflex for either quercetin or placebo (*p* > 0.05) (Table 1). RTD for quercetin was significantly decreased from PRE to POST (*p* < 0.05) (Table 1). A significant decrease in MVC from PRE to POST was noted with both quercetin and placebo (*p* < 0.05) (Table 1). There were no significant differences in T-reflex, RTD, MVC, twitch torques during singlet and doublet stimulations between quercetin and placebo ingestions at PRE and POST (*p* > 0.05) (Table 1). Duration between electrical stimulation and twitch torque for singlet stimulation were 76.1 ± 4.9 ms for quercetin at PRE, 76.5 ± 6.5 ms for quercetin at POST, 76.5 ± 5.7 ms for placebo at PRE, and 76.3 ± 6.1 ms for placebo at POST. There were no significant differences between PRE and POST for quercetin and placebo groups and between quercetin and placebo groups at PRE and POST (*p* > 0.05). RTD during electrically elicited contraction for singlet stimulation were 0.70 ± 0.19 Nm/ms for quercetin at PRE, 0.67 ± 0.20 Nm/ms for quercetin at POST, 0.70 ± 0.17 Nm/ms for placebo at PRE, and 0.68 ± 0.15 Nm/ms for placebo at POST. While there were no significant differences between PRE and POST for quercetin and placebo groups (*p* > 0.05), significant decrease was observed from PRE to POST in quercetin (*p* < 0.05), but not in placebo (*p* > 0.05).

**Table 1:** Results of measurements for neuromuscular performances before and after quercetin and placebo ingestions. PRE, before the ingestions; POST, 60 min after the ingestions

	Quercetin		Placebo		Q vs P
	Mean ± SD	Pre vs Post	Mean ± SD	Pre vs Post	
T-reflex					
Pre (mV)	0.055 ± 0.035		0.056 ± 0.041		*p* = 0.807
Post (mV)	0.061 ± 0.047	*p * = 0.917	0.074 ± 0.062	*p* = 0.087	*p* = 0.600
Post/Pre (%)	128.2 ± 67.1		124.7 ± 46.4		*p* = 0.861
RTD					
Pre (Nm/s)	2153 ± 819		2169 ± 913		*p* = 0.972
Post (Nm/s)	1909 ± 615	*p* = 0.033	1975 ± 661	*p* = 0.463	*p* = 0.173
Post/Pre (%)	91.5 ± 14.9		97.7 ± 23.6		*p *= 0.224
MVC					
Pre (Nm)	230.4 ± 42.7		236.3 ± 47.3		*p* = 0.221
Post (Nm)	216.4 ± 35.0	*p = 0.028	223.5 ± 50.2	*p = 0.039	*p *= 0.311
Post/Pre (%)	93.9 ± 10.9		93.0 ± 12.2		*p* = 0.650
Singlet stimulation					
Pre (Nm)	52.8 ± 14.8		53.3 ± 12.2		*p* = 0.753
Post (Nm)	50.8 ± 15.8	*p* = 0.116	51.9 ± 11.9	*p = 0.039	*p* = 0.507
Post/Pre (%)	95.1 ± 9.8		97.5 ± 5.4		*p* = 0.382
Doublet stimulation					
Pre (Nm)	74.4 ± 18.2		77.1 ± 17.1		*p* = 0.221
Post (Nm)	72.1 ± 22.5	*p* = 0.087	73.9 ± 17.6	*p = 0.005	*p* = 0.249
Post/Pre (%)	95.0 ± 10.9		95.5 ± 5.4		*p* = 0.972

*p < 0.05 significant differences between PRE and POST

For electrically elicited contractions, currents needed to reach the maximal knee extension joint torque were 638 ± 77 and 646 ± 66 mA with quercetin and placebo ingestions. There were significant decreases in twitch torques during singlet and doublet stimulations from PRE to POST with placebo (*p* = 0.039, *r* = 0.572 and *p* = 0.005, *r* = 0.785), but not with quercetin (*p* > 0.05) (Table 1).

**Fig. 4:**
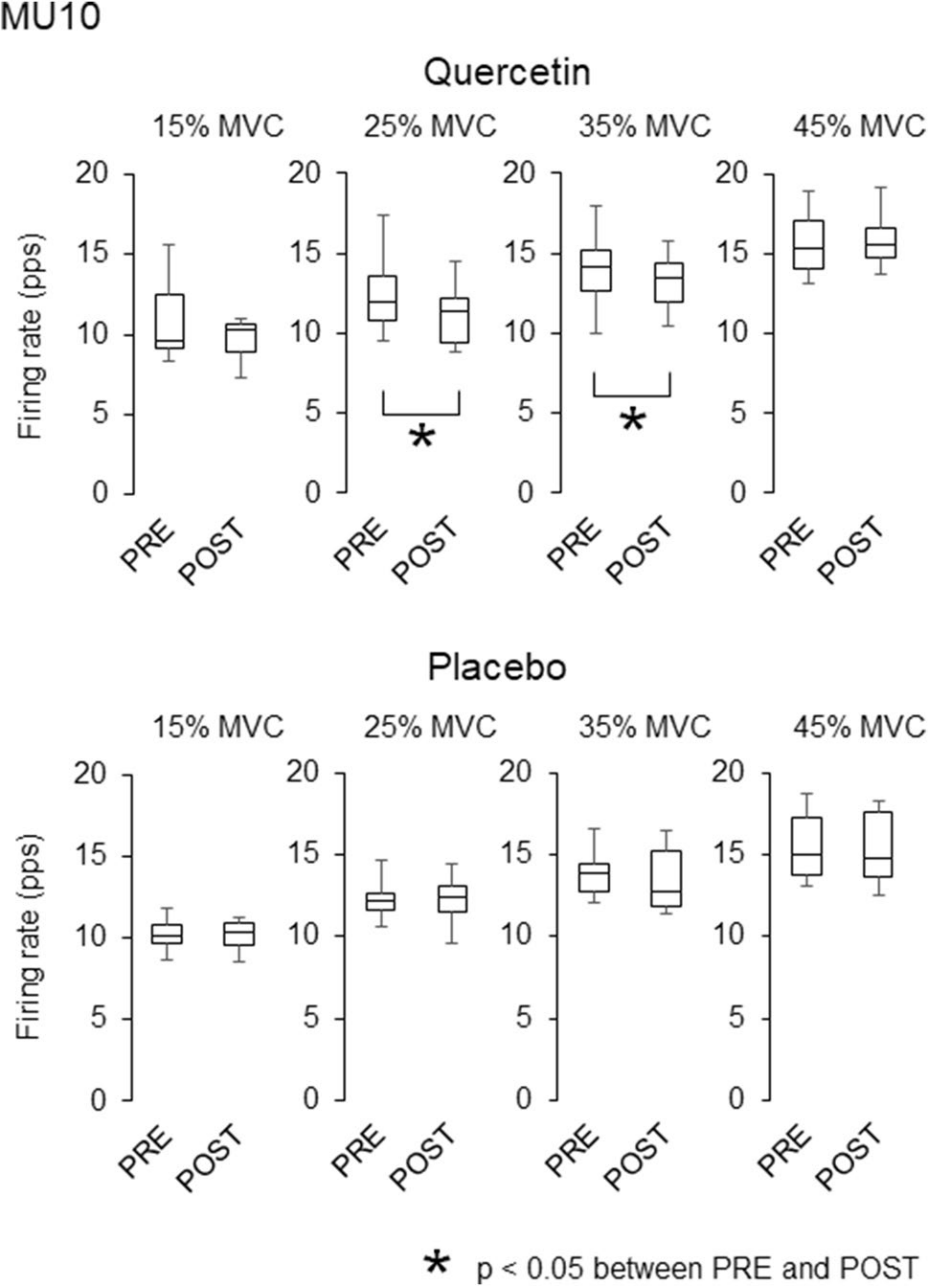
Motor unit firing rates during ramp contractions before and after quercetin (left) and placebo (right) ingestions for motor units recruited at < 15% of MVC (MU10). The lines within boxes, boxes, and bars indicate median values, 25 and 75% of quartile, and range of the data. * *p* < 0.05 between PRE and POST

**Fig. 5:**
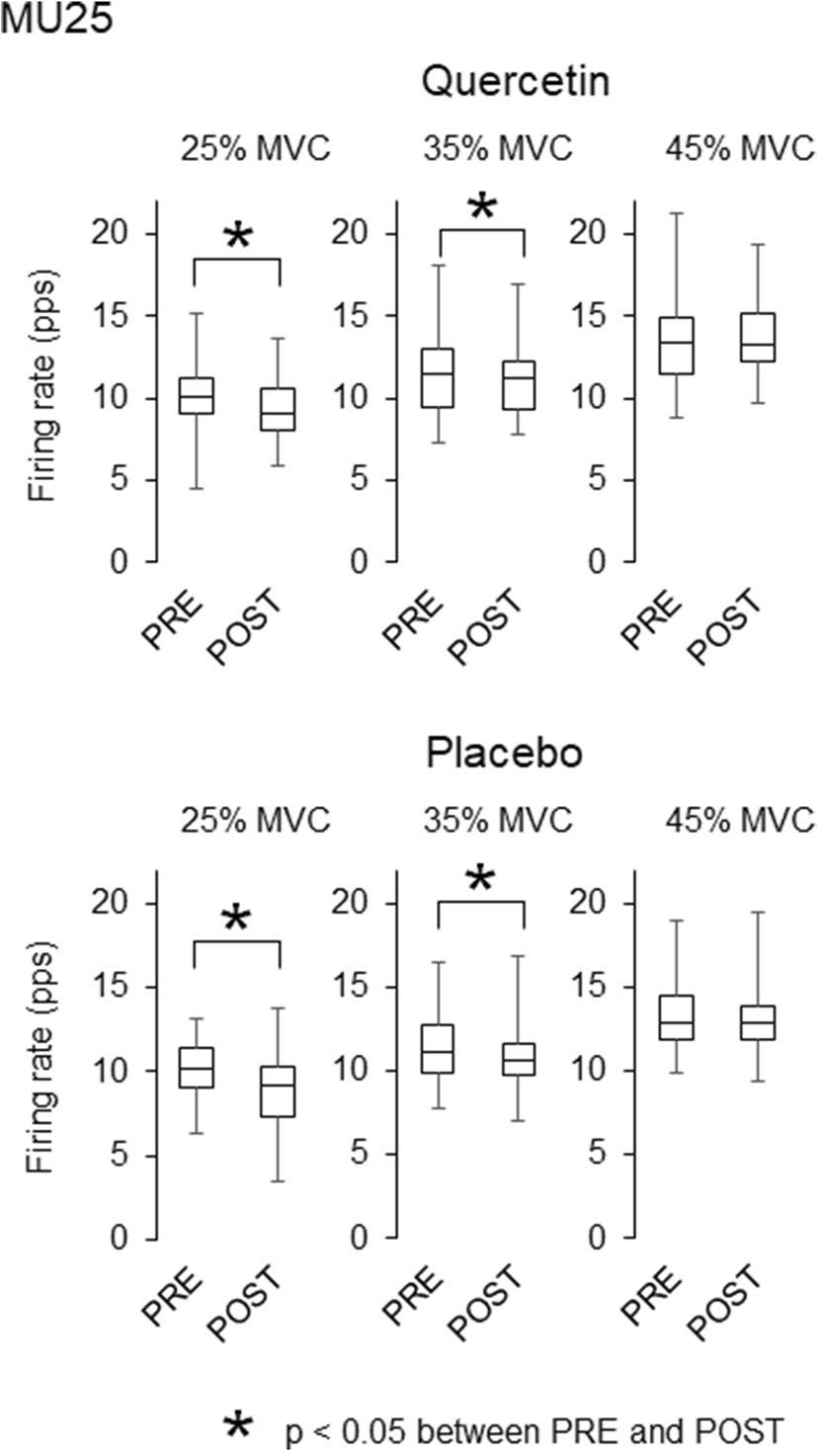
Motor unit firing rates during ramp contractions before and after quercetin (left) and placebo (right) ingestions for motor units recruited at 15–35% of MVC (MU25). The lines within boxes, boxes, and bars indicate median values, 25 and 75% of quartile, and range of the data. * *p* < 0.05 between PRE and POST

**Fig. 6:**
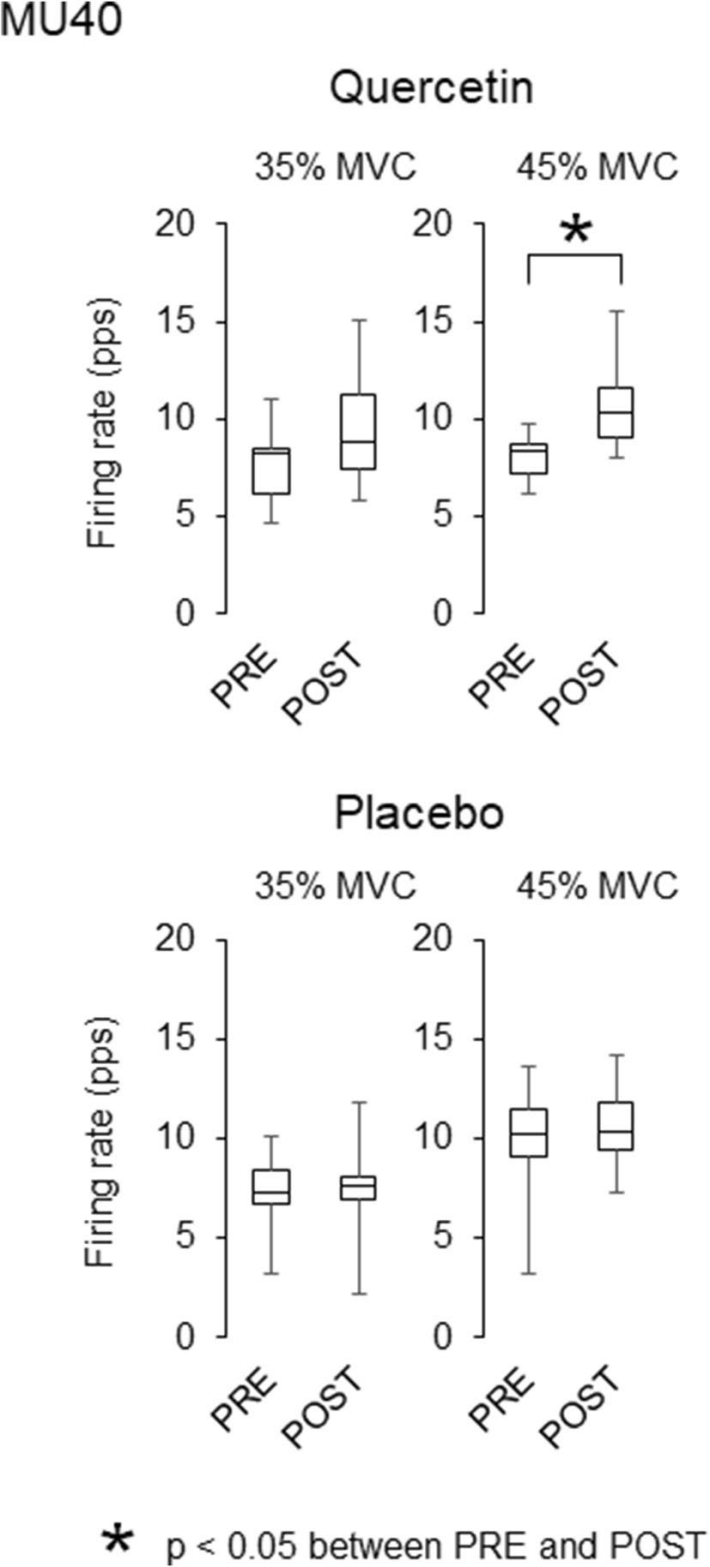
Motor unit firing rates during ramp contractions before and after quercetin (left) and placebo (right) ingestions for motor units recruited at 35–45% of MVC (MU40). The lines within boxes, boxes, and bars indicate median values, 25 and 75% of quartile, and range of the data. *p < 0.05 between PRE and POST

During ramp contractions, 90 and 99 motor units were detected with quercetin and placebo, respectively, and they were used for analysis (Table 2). Significant decreases in recruitment thresholds were observed in MU25 (*p* = 0.002, *r* = 0.457), MU40 (*p* = 0.009, *r* = 0.475), and all motor units (*p* = 0.003, *r* = 0.316) in quercetin ingestion (Table 3). There was significant decrease in recruitment threshold in MU40 (*p* = 0.001, *r* = 0.575) in placebo ingestion (Table 3). Significant decreases in the firing rate from PRE to POST were found for MU10 at 25 and 35% of MVC with quercetin (*p* = 0.015, *r* = 0.608 and p = 0.019, *r* = 0.588) (Fig. 4) and for MU25 at 25 and 35% of MVC in both quercetin (*p* = 0.011, *r* = 0.385 and *p* = 0.021, *r* = 0.347) and placebo (p = 0.004, *r* = 0.394 and *p* = 0.002, *r* = 0.413) (Fig. 5). A significant increase from PRE to POST in the firing rate for MU40 at 45% of MVC was observed with quercetin (*p* = 0.034, *r* = 0.386) (Fig. 6).

**Table 2:** Number of the analyzed motor units for merged data and individual participants

	Task	MU Group	Merged	Individual participants		
				Average	SD	Range
Quercetin	Ramp	MU10	16	1.2	1.7	0–5
		MU25	44	3.4	2.6	0–10
		MU40	30	2.3	2.4	0–7
		All	90	6.9	3.5	3–14
	Sustained	All	89	6.8	3.6	0–11
Placebo	Ramp	MU10	9	0.7	1.1	0–3
		MU25	55	4.2	3.4	0–12
		MU40	35	2.7	2.2	0–7
		All	99	7.6	4.5	2–19
	Sustained	All	90	6.9	4.4	0–15
				Number of motor units

**Table 3:** Recruitment thresholds of the analyzed motor units during ramp contractions

	PRE	POST	
Quercetin			
MU10	12.2 ± 2.1	14.4 ± 6.0	
MU25	25.9 ± 5.5	24.5 ± 6.7	*
MU40	39.5 ± 2.6	36.3 ± 6.0	*
All	28.3 ± 10.3	26.9 ± 9.9	*
Placebo			
MU10	10.8 ± 2.4	11.5 ± 2.2	
MU25	26.6 ± 5.8	26.9 ± 6.0	
MU40	38.8 ± 2.9	36.8 ± 3.7	*
All	29.7 ± 9.3	29.2 ± 8.6	
	(% of MVC)		

*p < 0.05 between PRE and POST

**Fig. 7:**
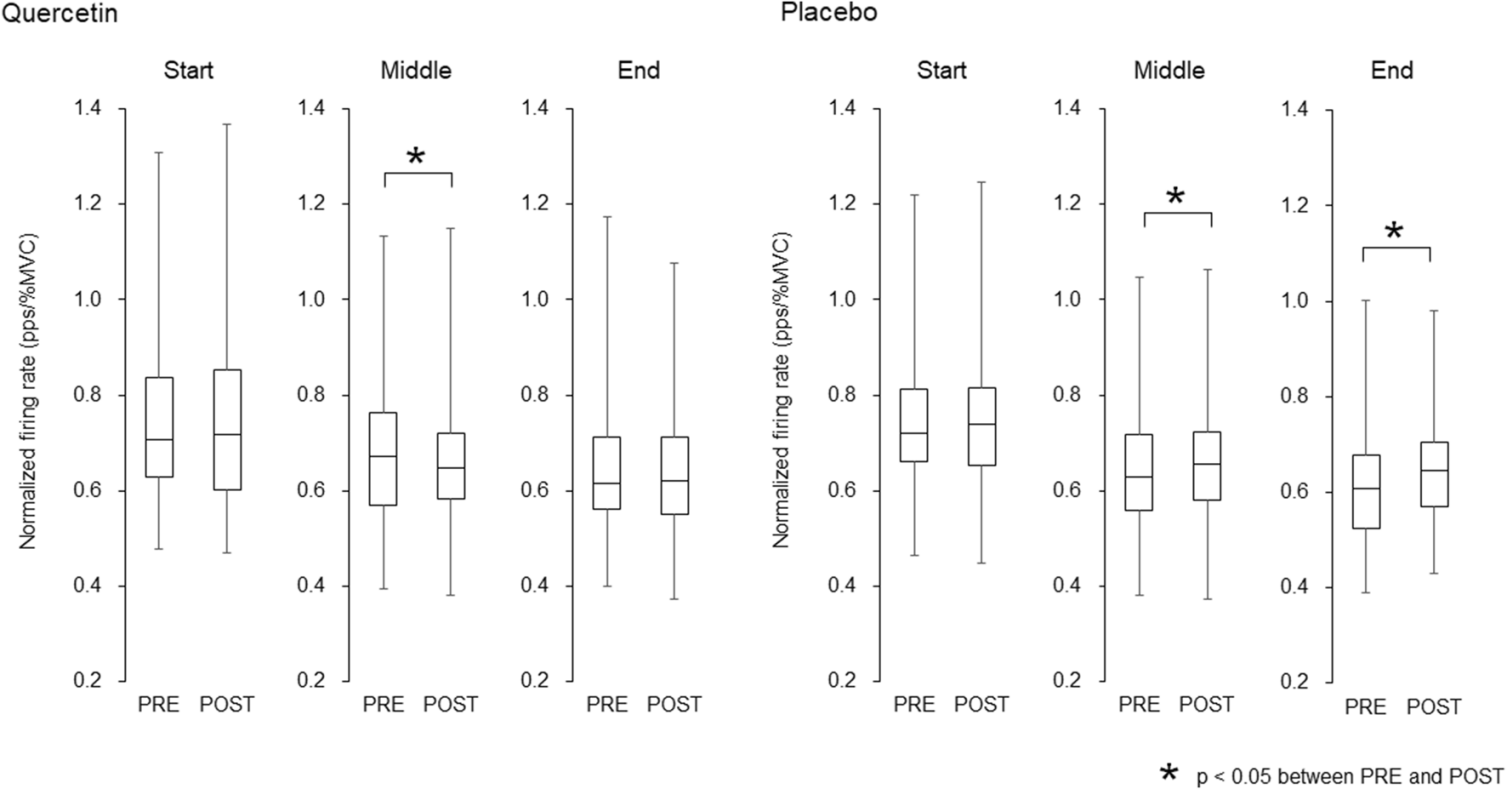
Normalized firing rate during sustained contractions before and after quercetin (left) and placebo (right) ingestions. PRE, before ingestions; POST, 60 min after ingestions. The lines within boxes, boxes, and bars indicate median values, 25 and 75% of quartile, and range of the data. *p < 0.05 between PRE and POST

During sustained contractions, 89 and 90 motor units were identified with quercetin and placebo, respectively, and they were used for analysis (Table 2). A significant decrease from PRE to POST in normalized firing rate at the middle was shown with quercetin (*p* = 0.005, *r* = 0.297) while increase in this parameter were seen at the middle and end in placebo (*p* = 0.010, *r* = 0.270 and *p* = 0.001, *r* = 0.507) (Fig. 7).

## Discussion

The present study investigated the acute effect of quercetin ingestion on motor unit firing patterns and muscle contractile properties. We found that quercetin ingestion leads to (1) absence of decrease in electrically elicited muscle contraction torques, (2) decreases and increases in firing rates of motor units recruited at lower and higher investigated forces, respectively, and (3) decreases in normalized firing rate. These findings support our hypotheses that quercetin ingestion induces alterations in the recruitment threshold-dependent motor unit firing pattern, and decrease in firing rate due to greater neuromuscular efficiency during voluntary contractions. The hypothesis that quercetin ingestion induces greater evoked twitch torques were rejected.

### Motor unit activations

While the present study revealed different firing rates of MU10 and MU40 between quercetin and placebo, firing rates in MU25 at 25 and 35% of MVC during ramp contractions were similarly decreased from PRE to POST with both quercetin and placebo ingestions (Figs. 4, 5, 6). Decreases in motor unit firing rates at the same absolute force are generally interpreted as adaptations that increase neuromuscular efficiency and/or reduced excitation from the central nervous system. Neuromuscular efficiency is strongly dependent on muscle contractile properties. We showed decreases in twitch torques during singlet and doublet stimulations from PRE to POST with placebo, but not with quercetin (Table 1). Also, normalized firing rate were decreased with quercetin and increased with placebo during sustained contractions (Fig. 7). Therefore, different physiological mechanisms may lead to the same results in MU25 with quercetin and placebo (Fig. 5). We consider that reductions in firing rates of MU10 and MU25 with quercetin (Figs. 4 and 5) may reflect acute adaptations in central nervous system from PRE to POST because of non-impaired muscle contractile properties (Table 1) and decrease in normalized firing rate during sustained contractions (Fig. 7). On the other hand, decreases in motor unit firing rates during ramp contractions with placebo (Fig. 5) could be explained by reductions of excitation from the central nervous system and possible recruitments of new motor units, which we were not able to cover in the present study. Indeed, in our study, we showed decreases in muscle contractile properties (Table 1) and increase in normalized firing rate with placebo (Fig. 7).

With quercetin ingestion, opposite responses from PRE to POST in firing rates were found between motor units recruited at relatively lower (MU10) and higher (MU40) force levels (Figs. 4 and 6). As discussed above, decreases in firing rates in MU10 could be associated with acute adaptations in central nervous system. Increases in the motor unit firing rate when the same absolute force is produced reflects adaptations to decreased neuromuscular efficiency and/or increased excitation from the central nervous system. Motor neurons with relatively higher and lower recruitment thresholds normally innervate muscle fibers that contribute to higher and lower forces, respectively (Henneman et al. 1965; Burke et al. 1973). Since alterations in neuromuscular efficiency would be induced by changes in muscle contractile properties, their different behaviors may be reasonable in the case of different changes in peripheral components of the neuromuscular system. Lopes et al. (1983) reported that increases in submaximal tension during electrically elicited muscle stimulation were selectively observed at a lower stimulation frequency in the range of 10–100 Hz following caffeine ingestion (Lopes et al. 1983). We thus inferred that the effect of caffeine ingestion on contractile properties may be induced in selective muscle fiber types. Since caffeine shares many similarities of its functions with quercetin regarding the effects on ionic processes of muscle contraction, we assumed that recruitment threshold-dependent changes in motor unit firing rates may be partly explained by alterations in muscle contractile properties in specific muscle fiber types.

Patrizio et al. (2018) and Bazzucchi et al. (2019) showed an increase in the median frequency of surface EMG (Patrizio et al. 2018) and a relative increase in muscle fiber conduction velocity (Bazzucchi et al. 2019) following quercetin ingestion, respectively. From these findings, they suggested the possibility that quercetin ingestion enhances the recruitment of motor units with higher recruitment thresholds. Similar to caffeine (McLellan et al. 2016), quercetin has been reported to function as an antagonist of A_1_ adenosine receptors (Alexander 2006) that can attenuate inhibitions of neurotransmitter release and neuronal firing rates. This action has been considered to alter arousal and induce excitations of the central nervous system following caffeine ingestion (Graham 2001; McLellan et al. 2016; Grgic et al. 2019). Our results showed a selective increase in firing rates of motor units recruited at higher investigated force levels (Figs. 4, 5, 6). We also found decreases in recruitment thresholds in MU25 and MU40 after quercetin ingestion and in MU40 for placebo (p < 0.05). It was reported that recruitment threshold of motor units is decreased with fatigue in vastus lateralis muscle (Adam and De Luca 2003). This study also showed no differences in recruitment threshold decline between high (> 25% MVC) and low (< 25% MVC) threshold motor units. On the other hand, Carpentier et al. (2001) showed no change and increase of recruitment threshold in low (< 25% MVC) threshold motor units and decrease of recruitment threshold in high (> 25% MVC) threshold motor units during fatiguing contraction for first dorsal interosseous muscle (Carpentier et al. 2001). Although it is difficult to identify the reason for selective decrease in recruitment threshold for specific motor unit groups in the present study, decrease in motor unit recruitment threshold from PRE to POST in placebo could be due to fatigue which was reflected as decrease MVC. This result shows that while decreases in recruitment thresholds were observed for both quercetin and placebo ingestions, quercetin ingestion provides decrease in recruitment thresholds for larger types of motor units. Although it is difficult to explain the detailed physiological pathways based on our data, these findings suggests the possibility of enhanced recruitment of larger numbers of motor units or high-threshold motor units by the ingestion of quercetins, which has also been postulated in the previous studies using quercetin (Patrizio et al. 2018; Bazzucchi et al. 2019) and caffeine (Graham 2001; McLellan et al. 2016; Grgic et al. 2019).

We found significant decrease in RTD from PRE to POST only for quercetin (*p* < 0.05) (Table 1). Considering that decrease in twitch forces from PRE to POST was observed only for placebo, decrease in RTD for quercetin could be partly explained by decline of force production ability in central nervous system. Therefore, we should note that alterations in motor unit firing properties by quercetin ingestion is not necessarily positive effects on maximal force production during ballistic/rapid contractions. However, we used constant statistic contraction for assessment of motor unit firing properties. Since motor unit firing properties would be different among different muscle contraction types (Enoka 1996; Van Cutsem et al. 1998; Duchateau and Enoka 2011), further studies are needed to clarify the effects of contraction type on the quercetin ingestion-induced adaptations in motor unit firing properties.

### Muscle contractile properties

Since quercetin has actions affecting calcium release from the sarcoplasmic reticulum (Lee et al. 2002), we hypothesized that quercetin ingestion induces greater evoked twitch torques. However, there were no significant differences in twitch torques during singlet and doublet stimulations between quercetin and placebo ingestions at POST (Table 1) and this hypothesis was rejected in the present study.

Although direct effects of quercetin ingestion on muscle contractile properties were not detected, interesting results were observed in changes in twitch torques during singlet and doublet stimulations from before to after quercetin and placebo ingestions. We showed significant decreases in twitch torques during singlet and doublet stimulations from PRE to POST with placebo (*p* < 0.05) (Table 1). Since MVC is also decreased from PRE to POST with either quercetin and placebo (*p* < 0.05) (Table 1), a decrease in electrically elicited contraction force means that our measurements show decreases in neuromuscular performance from PRE to POST. It is well known that muscle contractile properties is more sensitive to fatigue induced by voluntary contraction than central nervous system and decrease in MVC and electrically elicited twitch torque decrease even after single MVC trial (Bigland-Ritchie et al. 1986). Due to the repeated maximal and submaximal contractions, i.e., ten RTD, two MVC, and submaximal ramp contractions, decreased motor performance and/or attenuation of excitation in neuromuscular system may be induced in POST measurements and can partly explain alterations in muscle contractile properties with placebo. In fact, Bazzucchi et al. (2011) also demonstrated reduction of evoked twitch torque and MVC for elbow flexor muscles 60 min after placebo ingestion in their study which tested the effect of caffeine ingestion (Bazzucchi et al. 2011). Electrically elicited twitch torque depends on potentiation, i.e., phosphorylation of the myosin light chain which is known to induce increased calcium sensitivity, and two-trains of stimulation at low frequency (10–20 Hz, 50–100 ms of pulse gap) is associated with a failure in the excitation–contraction coupling such as reduction in calcium release (Millet et al. 2011). Therefore, we thought that decrease in electrically elicited twitch torque in placebo is reasonable result and could be due to reduction in calcium release by fatigue which is induced by the given tasks in the present study. On the other hand, decreases in twitch torques during singlet and doublet stimulations from PRE to POST were not found with quercetin (*p* > 0.05) (Table 1). One of the possible mechanisms of impairment of peripheral components such as a decrease in the twitch torque involves alterations in excitation and contraction (E-C) coupling. Generally, a reduction of calcium release from the sarcoplasmic reticulum occurs after repeated muscle contractions, impairing E-C coupling and decreasing contractile force productions (Cairns and Lindinger 2008). We thus considered that the decrease in twitch torques with placebo in the present study would be partly caused by this ionic alteration, and the absence of a decrease in twitch torques with quercetin could be explained by the increase in calcium release from the sarcoplasmic reticulum due to quercetin (Lee et al. 2002). Patrizio et al. (2018) reported that while placebo ingestion led to decreased torque production following resistance training, quercetin ingestion led to no change in torque production, and there were no significant differences in changes in the surface EMG amplitude between placebo and quercetin ingestions (Patrizio et al. 2018). This result suggests that absence of a reduction in torque production after quercetin ingestion could be associated with its actions on peripheral components such as muscle contractile properties (Lee et al. 2002). From the findings in the present and previous studies (Patrizio et al. 2018), we suggest that quercetin ingestion exhibits acute adaptations in peripheral components of the neuromuscular system by its effect on calcium release on E-C coupling (Lee et al. 2002). Previous studies that showed a positive effect of caffeine ingestion on muscle contractile properties (Lopes et al. 1983; Bazzucchi et al. 2011) may support our interpretation, since caffeine also has the physiological function to increase calcium release from the sarcoplasmic reticulum (Lee et al. 2002).

We found no significant changes in duration between electrical stimulation and occurrence of twitch torque from PRE to POST for quercetin and placebo and no significant differences between quercetin and placebo at PRE and POST in duration between electrical stimulation and twitch torque (*p* > 0.05). This means that the time course between electrical and mechanical events were not affected by quercetin ingestion. However, significant decreases in RTD were observed during electrically elicited contractions from PRE to POST for quercetin (*p* < 0.05), but not for placebo (*p* > 0.05) (Table 1). Since decrease in peak twitch torques from PRE to POST was observed only for placebo, it seems that quercetin ingestion provides different adaptations in peak force capacity and force development process during electrically elicited contraction. These findings suggest that the quercetin ingestions may induce delayed onset of muscle contraction, while it could mitigate fatigue-induced reduction in the muscle contractile force capacity.

### Limitation

In the present study, the detected motor units from different participants were merged for each condition (Quercetin/Placebo) for further analysis. Since we prioritized tracking of same motor units between PRE and POST, numbers of analyzed motor units were limited for a participant and varied among the participants (Table 2). For ramp contractions, the detected motor units were divided into three groups by recruitment thresholds for analysis the effect of recruitment threshold on motor unit firing pattern. The merged data showed the recruitment threshold-dependent alterations in motor unit firing rates following quercetin ingestion. However, we could not confirm this results in individual participants, because it was difficult to collect motor units from all three different recruitment threshold groups across two conditions (Quercetin/Placebo) in individual participants. This is major limitation of the present study and should be considered to interpret our results. Also, the motor unit grouping in the present study was based on recruitment threshold. Therefore, we need to note that the results of the present study from different motor unit groups during ramp contractions may not reflect physiological meanings of different types of motor units.

## Conclusion

We tested the acute effect of quercetin ingestion on the motor unit firing rate and muscle contractile properties in the human neuromuscular system. Quercetin ingestion led to the absence of decrease in electrically elicited muscle contraction forces following the given multiple measurements, i.e., tendon reflex, RTD, MVC, submaximal contractions, and electrical stimulations, and/or 60 min of resting, increases in firing rates of motor units recruited at higher investigated force levels, and decreases in normalized firing rate during sustained contractions at low force levels. From these findings, we suggest that quercetin ingestion plays a functional role to mitigate reduction in the muscle contractile properties, enhance activations of higher threshold motor units, and inhibit activation of lower threshold motor units. These effects can be explained by the physiological actions of quercetin to increase calcium release from the sarcoplasmic reticulum (Lee et al. 2002) and block the binding of adenosine to A_1_ receptors (Alexander 2006). We conclude that quercetin ingestion alters central and peripheral components of the human neuromuscular system and may be useful to improve neuromuscular performance.

## Data Availability

Data available on request from the authors upon reasonable request.
