# Correction to: Quercetin ingestion modifies human motor unit firing patterns and muscle contractile properties

**DOI:** 10.1007/s00221-021-06133-5

**Published:** 2021-07-27

**Authors:** Kohei Watanabe, Aleš Holobar

**Affiliations:** 1 Laboratory of Neuromuscular Biomechanics, Faculty of Liberal Studies and Sciences and School of International Liberal Studies, Chukyo University, Showa-ku, Yagotohonmachi, Nagoya 466-8666 Japan; 2 Faculty of Electrical Engineering and Computer Science, University of Maribor, Maribor, Slovenia

## Correction to: Experimental Brain Research (2021) 239:1567–1579 10.1007/s00221-021-06085-w

The article: Quercetin ingestion modifies human motor unit firing patterns and muscle contractile properties, written by Kohei Watanabe, Aleš Holobar was originally published electronically on the publisher’s internet portal (currently SpringerLink) on Published online: 19 March 2021 without open access.

With the author(s)’ decision to opt for Open Choice the copyright of the article changed on 10 May 2021 © The Author(s) 2021 and the article is forthwith distributed under the terms of the Creative Commons Attribution 4.0 International License (http://creativecommons.org/licenses/by/4.0/), which permits use, duplication, adaptation, distribution and reproduction in any medium or format, as long as you give appropriate credit to the original author(s) and the source, provide a link to the Creative Commons license and indicate if changes were made.

**Open Access** This article is distributed under the terms of the Creative Commons Attribution 4.0 International License (http://creativecommons.org/licenses/by/4.0/), which permits unrestricted use, distribution, and reproduction in any medium, provided you give appropriate credit to the original author(s) and the source, provide a link to the Creative Commons license, and indicate if changes were made.

The original article was corrected.

